# Common plants as alternative analytical tools to monitor heavy metals in soil

**DOI:** 10.1186/1752-153X-6-S2-S6

**Published:** 2012-05-02

**Authors:** Daniela Malizia, Antonella Giuliano, Giancarlo Ortaggi, Andrea Masotti

**Affiliations:** 1 Chemistry Department, Sapienza University of Rome, P.le A.Moro 5, 00185 Rome, Italy

## Abstract

**Background:**

Herbaceous plants are common vegetal species generally exposed, for a limited period of time, to bioavailable environmental pollutants. Heavy metals contamination is the most common form of environmental pollution. Herbaceous plants have never been used as natural bioindicators of environmental pollution, in particular to monitor the amount of heavy metals in soil. In this study, we aimed at assessing the usefulness of using three herbaceous plants (*Plantago major L.*, *Taraxacum officinale L.* and *Urtica dioica L.*) and one leguminous (*Trifolium pratense L.*) as alternative indicators to evaluate soil pollution by heavy metals.

**Results:**

We employed Inductively Coupled Plasma Atomic Emission Spectroscopy (ICP-AES) to assess the concentration of selected heavy metals (Cu, Zn, Mn, Pb, Cr and Pd) in soil and plants and we employed statistical analyses to describe the linear correlation between the accumulation of some heavy metals and selected vegetal species. We found that the leaves of *Taraxacum officinale L*. and *Trifolium pratense L.* can accumulate Cu in a linearly dependent manner with *Urtica dioica L*. representing the vegetal species accumulating the highest fraction of Pb.

**Conclusions:**

In this study we demonstrated that common plants can be used as an alternative analytical tool for monitoring selected heavy metals in soil.

## Background

Heavy metals contamination is one of the major kind of environmental pollution in urbanized cities due to emissions from heating, transport, industry and other human activities. In the past, the main contribution to heavy metals contamination has been due to lead used as anti detonating agent in fuels. At the end of 1998, the European Parliament and Council with the Directive 98/70/EC prohibited the marketing of leaded petrol within their territory. Since that date, the contribution of lead to heavy metal pollution have to depend from other anthropogenic sources (i.e., exausted batteries, paintings and other industrial wastes). Cadmium, zinc and nickel originate from oils, pneumatics and old car pieces in general, copper from cars and other electric vehicles and manganese prevalently from natural sources. Accumulation (and distribution) of anthropogenic heavy metals in soil may depend on wet and dry depositions that convey particles from air to soil. Heavy metals may impair plant physiology by reducing respiration and growth, interfering with photosynthetic processes and inhibiting fundamental enzymatic reactions if accumulated at high concentrations. When these toxic metals are present in soil at a low concentration, plants continue to grow uniformly despite accumulating these metals. The ability of plants to accumulate heavy metals into their organs may hence be used to monitor soil pollution, and in particular the amount of heavy metals.

In the past, several authors investigated the distribution of heavy metals in roadside soil [1-4], grass [5] and leaves [6,7] emphasizing lead accumulation in soils and vegetation [8-10], near highways [11], in small mammals [12,13], humans [14] and invertebrates [15,16]. Other authors focused their attention on heavy metals accumulation by higher plants in order to study the urban pollution [17-20].

One interesting study on the air pollution by vehicular traffic in Rome was reported [21], but only higher plants have been considered as environmental pollution markers.

In this study, common plants have been considered for two reasons. First, they are ephemeral: they live for a short time and thus they are exposed only for a very specific period of time to bioavailable pollutants. Second, they can be picked up more easily than other higher plants. Therefore, we studied three herbaceous plants (*Plantago major*, *Linnaeus*, *Taraxacum officinale* , *Linnaeus* and *Urtica dioica*, *Linnaeus*) and one leguminous (*Trifolium pratense*, *Linnaeus*) and we compared the heavy metals accumulation in roots and leaves. Together with Cu, Zn, Mn, and Pb we decided to consider also Cr and Pd to investigate if a significant release from vehicles components or from catalytic converters can occur. Our study is therefore aimed at finding simple and reliable vegetal indicators to monitor environmental pollution and in particular soil pollution by heavy metals.

## Experimental

### Reagents

Concentrated HNO_3_ (65%) was purchased by Sigma-Aldrich. Standard reference materials (SRM No. 2587 and 2711) were from the National Institute of Standards and Technology, Gaithersburg, USA.

### Apparatus

#### Analytical determination and data elaboration

The concentration of selected heavy metals (Cu, Mn, Zn, Pb, Cr and Pd) were determined by means of ICP-AES spectrophotometer (Varian Vista MPX CCD. Simultaneous ICP–OES) equipped with a U5000 AT+ nebulizer (Cetac Technologies). In order to maximize the element sensitivity and to avoid interferences, wavelengths were accurately chosen (324.754 nm for Cu, 257.610 nm for Mn, 206.200 nm for Zn, 220.353 for Pb, 267.716 for Cr and 340.458 for Pd) and two spectral regions were investigated. To assure a correct calibration of the instrument, at least one standard sample has been run every 10 test samples. Concentrations have been reported as mean values of three replicates. We found that all analytical determinations performed by ICP-MS are affected by an error equal to 5%. Data and graphics were elaborated with SigmaPlot Ver. 8.0 and Excel.

### Methods and procedures

#### Soil and plants sampling

For this study we considered four different vegetal species (*Plantago major L.*, *Taraxacum officinale L.*, *Urtica dioica L.* and *Trifolium pratense L.*) collected in spring (mid-March), in summer (at the end of June) and in autumn (beginning of October) of year 1999. Five sampling areas (SAs) in the city of Rome have been chosen according to their different level of anthropogenic pollution. In particular, two of these sites (SA1 and SA2) are located close to high-traffic roads (Muro Torto and Olimpica), other two near medium- and low- traffic (SA3 and SA4) roads (Ostiense and Eur) and the last (SA5) from a large park (Pamphili). The latter was assumed as the reference (uncontaminated) site.

Surface soils and plants samples (each weighing about 500 g) were taken in triplicate, at the same distance from the street across a 1x1 m^2^ area by employing a stainless steel trowel to a 20 cm depth from the surface. After classification, plants and surface soil samples have been put in suitable plastic containers on the same occurrence.

### Sample preparation and digestion procedure

Soil samples coming from the same site were pooled together, air-dried up to dryness, then sieved by passing through a 1 mm nylon sieve; fractions less than 1 mm size were further ground in an agate mortar, till all the sample was homogenized. Soil samples (particle size around 0.2 mm) were sealed in polyethylene bottles and stored.

The roots and leaves of the collected plants, suitably separated, were repeatedly washed first with tap water then with deionized water and finally air-dried. Roots samples from each of the three plants (of the same species) were pooled together, oven dried (105 °C, 48 h) homogenized and grinded in a metal free mill to obtain a fine powder. The same protocol was applied also to leaves.

For analysis, 350-400 mg (exactly weighted) of soil, roots or leaves were digested with 10 ml of concentrated HNO_3_ (65%) for 24 h at 130 °C in 25 ml round bottomed flasks equipped with reflux condensers. The vessels were cooled, and stock solutions were obtained by transferring samples in 25 ml volumetric flasks and made up to the mark with deionized water (0.05 μScm^-1^). The solution was filtered through a Whatman 541 paper and stored in glass bottles. Working solutions were obtained by diluting 1:10 (v:v) the correspondent stock solutions. Moreover, we performed also the analysis of blanks (clean mineralization solution) and standard reference materials (SRM) from the National Institute of Standards and Technology, Gaithersburg, USA (SRM No. 2587 and No. 2586 - Trace Elements in Soil containing lead from paint) in the same experimental conditions and by using the same protocol. The recovery varied from 95 to 98% and all the obtained values ±3σ were within the range of certified values.

## Results

### Analytical determinations

The mean concentration of Cu, Mn, Zn, Pb, Cr and Pd from surface soil, *Plantago major L.*, *Taraxacum officinale L.*, *Urtica dioica L.* and *Trifolium pratense L.* (both roots and leaves) have been summarized in Tables 1, 2, 3, 4.

**Table 1:** **Heavy metals concentrations in Plantago major L.** Cu, Mn, Zn, Pb, Cr and Pd soil, roots and leaves concentrations (ppm) in *Plantago major L.*

	SPRING			SUMMER			AUTUMN		
**Cu**	*Soil*	*Roots*	*Leaves*	*Soil*	*Roots*	*Leaves*	*Soil*	*Roots*	*Leaves*

SA1	111 ± 5.6	53 ± 2.7	17 ± 0.9	199 ± 10.0	124 ± 6.2	60 ± 3	188 ± 9.4	67 ± 3.4	37 ± 1.9
SA2	126 ± 6.3	62 ± 3.1	35 ± 1.8	214 ± 10.7	57 ± 2.9	26 ± 1.3	195 ± 9.8	104 ± 5.2	20 ± 1
SA3	52 ± 2.6	20 ± 1	10 ± 0.5	137 ± 6.9	93 ± 4.7	36 ± 1.8	129 ± 6.5	90 ± 4.5	29 ± 1.5
SA4	39 ± 2.0	30 ± 1.5	21 ± 1.1	93 ± 4.7	49 ± 2.5	18 ± 0.9	74 ± 3.7	67 ± 3.4	37 ± 1.9
SA5	27 ± 1.4	23 ± 1.2	20 ± 1	52 ± 2.6	39 ± 2.0	42 ± 2.1	43 ± 2.2	50 ± 2.5	39 ± 2.0

**Mn**	*Soil*	*Roots*	*Leaves*	*Soil*	*Roots*	*Leaves*	*Soil*	*Roots*	*Leaves*

SA1	773 ± 38.7	129 ± 6.5	51 ± 2.6	570 ± 28.5	160 ± 8	84 ± 4.2	627 ± 31.4	62 ± 3.1	31 ± 1.6
SA2	730 ± 36.5	72 ± 3.6	29 ± 1.5	509 ± 25.5	94 ± 4.7	34 ± 1.7	534 ± 26.7	106 ± 5.3	21 ± 1.1
SA3	784 ± 39.2	62 ± 3.1	72 ± 3.6	579 ± 29.0	147 ± 7.4	44 ± 2.2	579 ± 29.0	36 ± 1.8	29 ± 1.5
SA4	820 ± 41	171 ± 8.6	92 ± 4.6	560 ± 28	130 ± 6.5	56 ± 2.8	600 ± 30	113 ± 5.7	51 ± 2.6
SA5	745 ± 37.3	48 ± 2.4	33 ± 1.7	449 ± 22.5	80 ± 4	23 ± 1.2	552 ± 27.6	45 ± 2.3	35 ± 1.8

**Zn**	*Soil*	*Roots*	*Leaves*	*Soil*	*Roots*	*Leaves*	*Soil*	*Roots*	*Leaves*

SA1	206 ± 10.3	106 ± 5.3	57 ± 2.9	303 ± 15.2	199 ± 10.0	95 ± 4.8	342 ± 17.1	212 ± 10.6	121 ± 6.1
SA2	226 ± 11.3	158 ± 7.9	91 ± 4.6	321 ± 16.1	167 ± 8.4	75 ± 3.8	334 ± 16.7	156 ± 7.8	75 ± 3.8
SA3	104 ± 5.2	72 ± 3.6	51 ± 2.6	290 ± 14.5	134 ± 6.7	76 ± 3.8	368 ± 18.4	181 ± 9.1	95 ± 4.8
SA4	98 ± 4.9	75 ± 3.8	49 ± 2.5	240 ± 12	104 ± 5.2	51 ± 2.6	290 ± 14.5	154 ± 7.7	83 ± 4.2
SA5	71 ± 3.6	37 ± 1.9	39 ± 2.0	122 ± 6.1	90 ± 4.5	61 ± 3.1	116 ± 5.8	73 ± 3.7	68 ± 3.4

**Pb**	*Soil*	*Roots*	*Leaves*	*Soil*	*Roots*	*Leaves*	*Soil*	*Roots*	*Leaves*

SA1	578 ± 28.9	54 ± 2.7	12 ± 0.6	840 ± 42	35 ± 1.8	11 ± 0.6	686 ± 34.3	28 ± 1.4	8 ± 0.4
SA2	488 ± 24.4	38 ± 1.9	n.d.	792 ± 39.6	6 ± 0.3	n.d.	596 ± 29.8	15 ± 0.8	3 ± 0.2
SA3	276 ± 13.8	n.d.	n.d.	546 ± 27.3	26 ± 1.3	n.d.	523 ± 26.2	18 ± 0.9	3 ± 0.2
SA4	219 ± 11.0	n.d.	n.d.	425 ± 21.3	16 ± 0.8	n.d.	121 ± 6.1	10 ± 0.5	4 ± 0.2
SA5	137 ± 6.9	n.d.	n.d.	58 ± 2.9	2 ± 0.1	n.d.	82 ± 4.1	4 ± 0.2	n.d.

**Cr**	*Soil*	*Roots*	*Leaves*	*Soil*	*Roots*	*Leaves*	*Soil*	*Roots*	*Leaves*

SA1	40 ± 2	8 ± 0.4	2 ± 0.1	24 ± 1.2	3 ± 0.2	1 ± 0.1	26 ± 1.3	6 ± 0.3	1 ± 0.1
SA2	43 ± 2.2	9 ± 0.5	3 ± 0.2	21 ± 1.1	3 ± 0.2	1 ± 0.1	30 ± 1.5	3 ± 0.2	n.d.
SA3	33 ± 1.7	3 ± 0.2	1 ± 0.1	29 ± 1.5	5 ± 0.3	2 ± 0.1	33 ± 1.7	2 ± 0.1	n.d.
SA4	35 ± 1.8	7 ± 0.4	2 ± 0.1	27 ± 1.4	6 ± 0.3	1 ± 0.1	22 ± 1.1	3 ± 0.2	n.d.
SA5	22 ± 1.1	3 ± 0.2	1 ± 0.1	11 ± 0.6	4 ± 0.2	n.d.	16 ± 0.8	1 ± 0.1	n.d.

**Pd**	*Soil*	*Roots*	*Leaves*	*Soil*	*Roots*	*Leaves*	*Soil*	*Roots*	*Leaves*

SA1	71 ± 3.6	7 ± 0.4	3 ± 0.2	74 ± 3.7	3 ± 0.2	1 ± 0.1	72 ± 3.6	5 ± 0.3	1 ± 0.1
SA2	70 ± 3.5	7 ± 0.4	2 ± 0.1	72 ± 3.6	4 ± 0.2	2 ± 0.1	77 ± 3.9	6 ± 0.3	1 ± 0.1
SA3	73 ± 3.7	4 ± 0.2	1 ± 0.1	70 ± 3.5	4 ± 0.2	1 ± 0.1	74 ± 3.7	5 ± 0.3	1 ± 0.1
SA4	67 ± 3.4	7 ± 0.4	2 ± 0.1	73 ± 3.7	5 ± 0.3	2 ± 0.1	67 ± 3.4	7 ± 0.4	2 ± 0.1
SA5	41 ± 2.1	2 ± 0.1	1 ± 0.1	41 ± 2.1	3 ± 0.2	1 ± 0.1	44 ± 2.2	3 ± 0.2	1 ± 0.1

**Table 2:** **Heavy metals concentrations in Taraxacum officinale L.** Cu, Mn, Zn, Pb, Cr and Pd soil, roots and leaves concentrations (ppm) in *Taraxacum officinale L*.

	SPRING			SUMMER			AUTUMN		
**Cu**	*Soil*	*Roots*	*Leaves*	*Soil*	*Roots*	*Leaves*	*Soil*	*Roots*	*Leaves*

SA1	126 ± 6.3	36 ± 1.8	35 ± 1.8	136 ± 6.8	95 ± 4.8	51 ± 2.6	131 ± 6.6	97 ± 4.9	52 ± 2.6
SA2	116 ± 5.8	46 ± 2.3	39 ± 2.0	144 ± 7.2	64 ± 3.2	42 ± 2.1	142 ± 7.1	71 ± 3.6	45 ± 2.3
SA3	110 ± 5.5	31 ± 1.6	37 ± 1.9	155 ± 7.8	55 ± 2.8	39 ± 2.0	99 ± 5.0	27 ± 1.4	34 ± 1.7
SA4	54 ± 2.7	40 ± 2	23 ± 1.2	127 ± 6.4	31 ± 1.6	26 ± 1.3	74 ± 3.7	30 ± 1.5	24 ± 1.2
SA5	32 ± 1.6	15 ± 0.8	15 ± 0.8	104 ± 5.2	31 ± 1.6	24 ± 1.2	55 ± 2.8	25 ± 1.3	10 ± 0.5

**Mn**	*Soil*	*Roots*	*Leaves*	*Soil*	*Roots*	*Leaves*	*Soil*	*Roots*	*Leaves*

SA1	809 ± 40.5	49 ± 2.5	39 ± 2.0	546 ± 27.3	61 ± 3.1	41 ± 2.1	624 ± 31.2	43 ± 2.2	19 ± 1.0
SA2	720 ± 36	115 ± 5.8	64 ± 3.2	576 ± 28.8	112 ± 5.6	35 ± 1.8	602 ± 30.1	61 ± 3.1	42 ± 2.1
SA3	755 ± 37.8	76 ± 3.8	74 ± 3.7	571 ± 28.6	83 ± 4.2	58 ± 2.9	583 ± 29.2	65 ± 3.3	38 ± 1.9
SA4	788 ± 39.4	91 ± 4.6	98 ± 4.9	600 ± 30	66 ± 3.3	51 ± 2.6	580 ± 29	104 ± 5.2	54 ± 2.7
SA5	646 ± 32.3	45 ± 2.3	44 ± 2.2	631 ± 31.6	86 ± 4.3	59 ± 3.0	624 ± 31.2	59 ± 3.0	32 ± 1.6

**Zn**	*Soil*	*Roots*	*Leaves*	*Soil*	*Roots*	*Leaves*	*Soil*	*Roots*	*Leaves*

SA1	229 ± 11.5	155 ± 7.8	133 ± 6.7	374 ± 18.7	211 ± 10.6	148 ± 7.4	742 ± 37.1	227 ± 11.4	90 ± 4.5
SA2	220 ± 11	157 ± 7.9	121 ± 6.1	426 ± 21.3	234 ± 11.7	150 ± 7.5	678 ± 33.9	265 ± 13.3	137 ± 6.9
SA3	215 ± 10.8	119 ± 6.0	109 ± 5.5	263 ± 13.2	152 ± 7.6	105 ± 5.3	694 ± 34.7	187 ± 9.4	64 ± 3.2
SA4	101 ± 5.1	94 ± 4.7	79 ± 4.0	254 ± 12.7	72 ± 3.6	80 ± 4	393 ± 19.7	73 ± 3.7	55 ± 2.8
SA5	61 ± 3.1	59 ± 3.0	70 ± 3.5	93 ± 4.7	70 ± 3.5	80 ± 4	140 ± 7	73 ± 3.7	40 ± 2

**Pb**	*Soil*	*Roots*	*Leaves*	*Soil*	*Roots*	*Leaves*	*Soil*	*Roots*	*Leaves*

SA1	627 ± 31.4	68 ± 3.4	8 ± 0.4	796 ± 39.8	109 ± 5.5	22 ± 1.1	730 ± 36.5	84 ± 4.2	11 ± 0.6
SA2	588 ± 29.4	75 ± 3.8	n.d.	769 ± 38.5	155 ± 7.8	28 ± 1.4	730 ± 36.5	108 ± 5.4	22 ± 1.1
SA3	206 ± 10.3	n.d.	n.d.	644 ± 32.2	26 ± 1.3	12 ± 0.6	560 ± 28	15 ± 0.8	3 ± 0.2
SA4	244 ± 12.2	n.d.	n.d.	371 ± 18.6	11 ± 0.6	8 ± 0.4	107 ± 5.4	9 ± 0.5	2 ± 0.1
SA5	148 ± 7.4	n.d.	n.d.	174 ± 8.7	6 ± 0.3	4 ± 0.2	89 ± 4.5	4 ± 0.2	1 ± 0.1

**Cr**	*Soil*	*Roots*	*Leaves*	*Soil*	*Roots*	*Leaves*	*Soil*	*Roots*	*Leaves*

SA1	35 ± 1.8	7 ± 0.4	3 ± 0.2	29 ± 1.5	8 ± 0.4	2 ± 0.1	30 ± 1.5	3 ± 0.2	1 ± 0.1
SA2	38 ± 1.9	10 ± 0.5	5 ± 0.3	34 ± 1.7	10 ± 0.5	3 ± 0.2	35 ± 1.8	4 ± 0.2	1 ± 0.1
SA3	40 ± 2	4 ± 0.2	3 ± 0.2	31 ± 1.6	7 ± 0.4	1 ± 0.1	32 ± 1.6	3 ± 0.2	1 ± 0.1
SA4	45 ± 2.3	7 ± 0.4	3 ± 0.2	29 ± 1.5	4 ± 0.2	2 ± 0.1	21 ± 1.1	n.d.	n.d.
SA5	33 ± 1.7	3 ± 0.2	3 ± 0.2	28 ± 1.4	2 ± 0.1	1 ± 0.1	28 ± 1.4	n.d.	n.d.

**Table 3:** **Heavy metals concentrations in Urtica dioica L.** Cu, Mn, Zn, Pb, Cr and Pd soil, roots and leaves concentrations (ppm) in *Urtica dioica L*.

	SPRING			SUMMER			AUTUMN		
**Cu**	*Soil*	*Roots*	*Leaves*	*Soil*	*Roots*	*Leaves*	*Soil*	*Roots*	*Leaves*

SA1	104 ± 5.2	42 ± 2.1	21 ± 1.1	186 ± 9.3	106 ± 5.3	41 ± 2.1	162 ± 8.1	51 ± 2.6	28 ± 1.4
SA2	92.5 ± 4.7	22 ± 1.1	19 ± 1.0	156 ± 7.8	100 ± 5	34 ± 1.7	169 ± 8.5	55 ± 2.8	39 ± 2.0
SA3	85 ± 4.3	18 ± 0.9	14 ± 0.7	105 ± 5.3	40 ± 2	15 ± 0.8	117 ± 5.9	61 ± 3.1	33 ± 1.7
SA4	38 ± 1.9	15 ± 0.8	17 ± 0.9	101 ± 5.1	56 ± 2.8	18 ± 0.9	105 ± 5.3	46 ± 2.3	23 ± 1.2
SA5	26 ± 1.3	17 ± 0.9	13 ± 0.7	47 ± 2.4	30 ± 1.5	20 ± 1	87 ± 4.4	51 ± 2.6	30 ± 1.5

**Mn**	*Soil*	*Roots*	*Leaves*	*Soil*	*Roots*	*Leaves*	*Soil*	*Roots*	*Leaves*

SA1	651 ± 32.6	158 ± 7.9	41 ± 2.1	580 ± 29	147 ± 7.4	71 ± 3.6	626 ± 31.3	163 ± 8.2	39 ± 2.0
SA2	603 ± 30.2	182 ± 9.1	37 ± 1.9	514 ± 25.7	104 ± 5.2	29 ± 1.5	554 ± 27.7	130 ± 6.5	34 ± 1.7
SA3	626 ± 31.3	121 ± 6.1	70 ± 3.5	532 ± 26.6	110 ± 5.5	63 ± 3.2	640 ± 32	169 ± 8.5	48 ± 2.4
SA4	587 ± 29.4	111 ± 5.6	54 ± 2.7	496 ± 24.8	86 ± 4.3	77 ± 3.9	620 ± 31	160 ± 8	78 ± 3.9
SA5	553 ± 27.7	98 ± 4.9	31 ± 1.6	518 ± 25.9	121 ± 6.1	69 ± 3.5	620 ± 31	142 ± 7.1	55 ± 2.8

**Zn**	*Soil*	*Roots*	*Leaves*	*Soil*	*Roots*	*Leaves*	*Soil*	*Roots*	*Leaves*

SA1	185 ± 9.3	97 ± 4.9	46 ± 2.3	255 ± 12.8	144 ± 7.2	128 ± 6.4	336 ± 16.8	198 ± 9.9	124 ± 6.2
SA2	137 ± 6.9	34 ± 1.7	19 ± 1.0	224 ± 11.2	103 ± 5.2	100 ± 5	428 ± 21.4	210 ± 10.5	152 ± 7.6
SA3	152 ± 7.6	49 ± 2.5	36 ± 1.8	150 ± 7.5	75 ± 3.8	65 ± 3.3	374 ± 18.7	172 ± 8.6	130 ± 6.5
SA4	160 ± 8	58 ± 2.9	41 ± 2.1	185 ± 9.3	91 ± 4.6	69 ± 3.5	223 ± 11.2	130 ± 6.5	98 ± 4.9
SA5	92 ± 4.6	26 ± 1.3	14 ± 0.7	147 ± 7.4	112 ± 5.6	95 ± 4.8	204 ± 10.2	145 ± 7.3	120 ± 6

**Pb**	*Soil*	*Roots*	*Leaves*	*Soil*	*Roots*	*Leaves*	*Soil*	*Roots*	*Leaves*

SA1	528 ± 26.4	75 ± 3.8	21 ± 1.1	888 ± 44.4	81 ± 4.1	23 ± 1.2	710 ± 35.5	95 ± 4.8	30 ± 1.5
SA2	452 ± 22.6	73 ± 3.7	19 ± 1.0	971 ± 48.6	60 ± 3	21 ± 1.1	854 ± 42.7	85 ± 4.3	23 ± 1.2
SA3	215 ± 10.8	44 ± 2.2	13 ± 0.7	548 ± 27.4	46 ± 2.3	14 ± 0.7	434 ± 21.7	59 ± 3.0	16 ± 0.8
SA4	152 ± 7.6	23 ± 1.2	11 ± 0.6	294 ± 14.7	37 ± 1.9	13 ± 0.7	230 ± 11.5	43 ± 2.2	14 ± 0.7
SA5	136 ± 6.8	32 ± 1.6	9 ± 0.5	202 ± 10.1	34 ± 1.7	10 ± 0.5	198 ± 9.9	37 ± 1.9	12 ± 0.6

**Cr**	*Soil*	*Roots*	*Leaves*	*Soil*	*Roots*	*Leaves*	*Soil*	*Roots*	*Leaves*

SA1	28 ± 1.4	5 ± 0.3	3 ± 0.2	36 ± 1.8	10 ± 0.5	5 ± 0.3	28 ± 1.4	6 ± 0.3	2 ± 0.1
SA2	26 ± 1.3	7 ± 0.4	4 ± 0.2	40 ± 2	12 ± 0.6	4 ± 0.2	30 ± 1.5	7 ± 0.4	3 ± 0.2
SA3	22 ± 1.1	5 ± 0.3	3 ± 0.2	43 ± 2.2	10 ± 0.5	4 ± 0.2	29 ± 1.5	4 ± 0.2	2 ± 0.1
SA4	21 ± 1.1	7 ± 0.4	5 ± 0.3	41 ± 2.1	11 ± 0.6	4 ± 0.2	27 ± 1.4	5 ± 0.3	2 ± 0.1
SA5	23 ± 1.2	5 ± 0.3	2 ± 0.1	25 ± 1.3	7 ± 0.4	3 ± 0.2	21 ± 1.1	4 ± 0.2	1 ± 0.1

**Pd**	*Soil*	*Roots*	*Leaves*	*Soil*	*Roots*	*Leaves*	*Soil*	*Roots*	*Leaves*

SA1	66 ± 3.3	12 ± 0.6	5 ± 0.3	65 ± 3.3	9 ± 0.5	3 ± 0.2	68 ± 3.4	13 ± 0.7	3 ± 0.2
SA2	71 ± 3.6	16 ± 0.8	9 ± 0.5	72 ± 3.6	10 ± 0.5	3 ± 0.2	72 ± 3.6	13 ± 0.7	3 ± 0.2
SA3	76 ± 3.8	16 ± 0.8	7 ± 0.4	70 ± 3.5	10 ± 0.5	2 ± 0.1	65 ± 3.3	10 ± 0.5	2 ± 0.1
SA4	67 ± 3.4	18 ± 0.9	9 ± 0.5	77 ± 3.9	11 ± 0.6	3 ± 0.2	70 ± 3.5	13 ± 0.7	3 ± 0.2
SA5	55 ± 2.8	8 ± 0.4	4 ± 0.2	57 ± 2.9	5 ± 0.3	1 ± 0.1	59 ± 3.0	6 ± 0.3	1 ± 0.1

**Table 4:** **Heavy metals concentrations in Trifolium pratense L.** Cu, Mn, Zn, Pb, Cr and Pd soil, roots and leaves concentrations (ppm) in *Trifolium pratense L*.

	SPRING			SUMMER			AUTUMN		
**Cu**	Soil	Roots	Leaves	Soil	Roots	Leaves	Soil	Roots	Leaves

SA1	93.1 ± 4.7	25.7 ± 1.3	20.5 ± 1.0	160.2 ± 8.0	103.3 ± 5.2	82.1 ± 4.1	126.4 ± 6.3	57.2 ± 2.9	26.5 ± 1.3
SA2	66.2 ± 3.3	21.3 ± 1.1	20.1 ± 1.0	132.5 ± 6.6	79.2 ± 4.0	57.8 ± 2.9	108.6 ± 5.4	80.1 ± 4.0	35.1 ± 1.8
SA3	55.1 ± 2.8	16.8 ± 0.8	10.3 ± 0.5	82.3 ± 4.1	31.5 ± 1.6	16.2 ± 0.8	65.6 ± 3.3	44.3 ± 2.2	23.5 ± 1.2
SA4	40.3 ± 2.0	21.5 ± 1.1	16.3 ± 0.8	97.7 ± 4.9	50.6 ± 2.5	27.3 ± 1.4	79 ± 4.0	46.5 ± 2.3	25.6 ± 1.3
SA5	36.1 ± 1.8	21.3 ± 1.1	11.2 ± 0.6	41.5 ± 2.1	35.4 ± 1.8	18.3 ± 0.9	49.3 ± 2.5	33.1 ± 1.7	19.8 ± 1.0

**Mn**	Soil	Roots	Leaves	Soil	Roots	Leaves	Soil	Roots	Leaves

SA1	640 ± 32	147.2 ± 7.4	39.3 ± 2.0	592 ± 29.6	166.3 ± 8.3	45.2 ± 2.3	597 ± 29.85	34 ± 1.7	63 ± 3.2
SA2	581 ± 29.1	104.5 ± 5.2	27.1 ± 1.4	527 ± 26.4	113.5 ± 5.7	37.8 ± 1.9	569 ± 28.5	115 ± 5.8	50 ± 2.5
SA3	608 ± 30.4	115.1 ± 5.8	32.1 ± 1.6	560 ± 28	128.7 ± 6.4	36.5 ± 1.8	600 ± 30	92 ± 4.6	78 ± 3.9
SA4	558 ± 27.9	100.2 ± 5.0	24.2 ± 1.2	503 ± 25.2	124.2 ± 6.2	31.2 ± 1.6	583 ± 29.1	67 ± 3.4	57 ± 2.9
SA5	510.7 ± 25.5	96.3 ± 4.8	21.8 ± 1.09	467.2 ± 23.4	114.1 ± 5.7	25.1 ± 1.3	548 ± 27.4	43 ± 2.2	61 ± 3.1

**Zn**	Soil	Roots	Leaves	Soil	Roots	Leaves	Soil	Roots	Leaves

SA1	251 ± 12.6	82.5 ± 4.1	43.2 ± 2.2	369 ± 18.5	130.2 ± 6.5	50.5 ± 2.5	407.2 ± 20.4	232 ± 11.6	141 ± 7.1
SA2	168.3 ± 8.4	79.2 ± 4.0	44.1 ± 2.2	336 ± 16.8	101.5 ± 5.1	49.1 ± 2.5	492.2 ± 24.6	289 ± 14.5	127 ± 6.4
SA3	188.3 ± 9.4	60.2 ± 3.0	37.1 ± 1.9	306 ± 15.3	99.2 ± 5.0	49.1 ± 2.5	461.2 ± 23.1	282 ± 14.1	91 ± 4.6
SA4	196.2 ± 9.8	61.3 ± 3.1	36.8 ± 1.8	290 ± 14.5	96.5 ± 4.8	41.2 ± 2.1	425.3 ± 21.265	94 ± 4.7	85 ± 4.3
SA5	126.5 ± 6.3	38.4 ± 1.9	25.6 ± 1.3	201.2 ± 10.1	85.2 ± 4.3	30.2 ± 1.5	255.1 ± 12.8	81 ± 4.1	73 ± 3.7

**Pb**	Soil	Roots	Leaves	Soil	Roots	Leaves	Soil	Roots	Leaves

SA1	698 ± 34.9	7 ± 0.35	15 ± 0.8	1163 ± 58.2	48 ± 2.4	6 ± 0.3	1051 ± 52.6	32 ± 1.6	12 ± 0.6
SA2	624 ± 31.2	0 ± 0	5 ± 0.3	1266 ± 63.3	58 ± 2.9	12 ± 0.6	1080 ± 54	28 ± 1.4	14 ± 0.7
SA3	236 ± 11.8	0 ± 0	11 ± 0.6	529 ± 26.5	9 ± 0.5	4 ± 0.2	490 ± 24.5	4 ± 0.2	9 ± 0.5
SA4	159 ± 8.0	0 ± 0	0 ± 0	349 ± 17.5	30 ± 1.5	4 ± 0.2	106 ± 5.3	10 ± 0.5	4 ± 0.2
SA5	126 ± 6.3	4 ± 0.2	0 ± 0	110 ± 5.5	10 ± 0.5	6 ± 0.3	80 ± 4	2 ± 0.1	2 ± 0.1

**Cr**	Soil	Roots	Leaves	Soil	Roots	Leaves	Soil	Roots	Leaves

SA1	25 ± 1.3	2 ± 0.1	0 ± 0	31 ± 1.6	3 ± 0.2	1 ± 0.1	30 ± 1.5	3 ± 0.2	3 ± 0.2
SA2	22 ± 1.1	4 ± 0.2	2 ± 0.1	37 ± 1.9	4 ± 0.2	2 ± 0.1	31 ± 1.6	3 ± 0.2	1 ± 0.1
SA3	26 ± 1.3	0 ± 0	1 ± 0.1	35 ± 1.8	0 ± 0	0 ± 0	25 ± 1.3	2 ± 0.1	1 ± 0.1
SA4	29 ± 1.5	3 ± 0.2	1 ± 0.1	31 ± 1.6	3 ± 0.2	2 ± 0.1	21 ± 1.1	2 ± 0.1	1 ± 0.1
SA5	22 ± 1.1	0 ± 0	2 ± 0.1	23 ± 1.2	0 ± 0	0 ± 0	15 ± 0.8	2 ± 0.1	1 ± 0.1

**Pd**	Soil	Roots	Leaves	Soil	Roots	Leaves	Soil	Roots	Leaves

SA1	70 ± 3.5	5 ± 0.3	0 ± 0	71 ± 3.6	5 ± 0.3	2 ± 0.1	68 ± 3.4	5 ± 0.3	2 ± 0.1
SA2	67 ± 3.4	4 ± 0.2	2 ± 0.1	68 ± 3.4	4 ± 0.2	2 ± 0.1	66 ± 3.3	4 ± 0.2	2 ± 0.1
SA3	69 ± 3.5	5 ± 0.3	0 ± 0	67 ± 3.4	4 ± 0.2	1 ± 0.1	60 ± 3	5 ± 0.3	1 ± 0.1
SA4	69 ± 3.5	5 ± 0.3	2 ± 0.1	60 ± 3	3 ± 0.2	1 ± 0.1	55 ± 2.8	4 ± 0.2	2 ± 0.1
SA5	37 ± 1.9	1 ± 0.1	0 ± 0	45 ± 2.3	2 ± 0.1	1 ± 0.1	47 ± 2.4	2 ± 0.1	1 ± 0.1

### Heavy metals in soil

We found that Cu, Mn, Zn, Pb, Cr and Pd amount in soil varies with the order SA1≈SA2>SA3>SA4 >SA5, being SA1 the most polluted area and SA5 the less contaminated one. Heavy metals concentration we found, is therefore closely linked to the level of contamination of the different sampling areas. The trend observed is independent on vegetal species considered and/or seasons. In every sampling site, among the heavy metals taken into consideration, Mn and Pb are the two most abundant whereas, Cr and Pd display the lowest concentrations.

**Figure 1:**
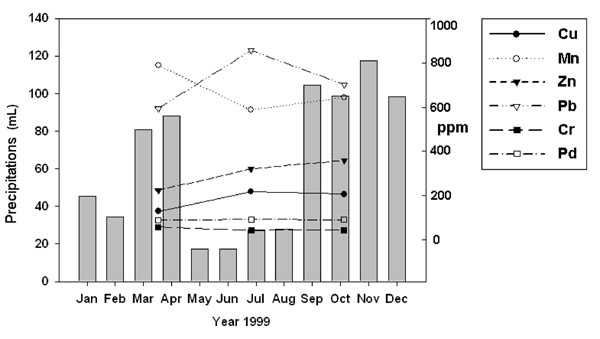
**Behaviour of heavy metals concentrations as a function of seasonal precipitations**. Cu, Mn, Zn, Pb, Cr and Pd concentrations (ppm) in spring, summer and autumn as a function of precipitations in Rome (Year 1999). As indicative example, metal concentrations were reported as mean values found in **SA1-SA5** soils in *Plantago major L.*

The results of the heavy metals determined in soil seems to evidence a seasonal dependence. Fig. 1 reports an indicative example of the seasonal variation of heavy metal concentration for Cu and Pb in *Plantago major L.* Concentrations of Cu and Pb reach the maximum value during summer while Mn reaches the minimum value. Zn concentration increases from spring to autumn while Cr and Pd concentrations remain relatively constant. Other factors can influence the local concentration of heavy metals in soil: temperature, rainfall, evapotranspiration, soil pH and redox potential. To correlate heavy metals concentration with the level of precipitation, we collected the rainfall data for the city of Rome from the Meteorological Centre of Rome. Superimposing the precipitations records with heavy metals concentrations we were able to observe some characteristic trends. In particular, during spring and autumn when the first and the third sampling occurred, moderate to abundant precipitation were registered whilst in summer rains are rare. The higher temperature and reduced rainfall may hence favour the water evaporation in soils leading to a higher accumulation of metals with respect to spring or autumn. Cu, and Pb seem to follow such a behaviour, with a maximum concentration during summer (214 ppm and 1266 ppm, respectively), while Zn concentration reaches a maximum during autumn (742 ppm). On the contrary, Mn follows the opposite trend showing the lowest value during summer (449 ppm). Cr and Pd seem not to be influenced by atmospheric conditions and their concentration remain relatively low and constant all over the year (between 15 and 45 ppm for Cr and between 37 and 77 ppm for Pd).

### Heavy metals in plants

**Figure 2:**
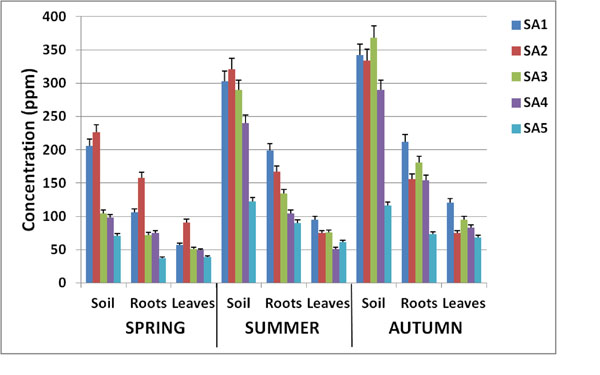
**Heavy metals concentration in soil, roots and leaves as a function of sampling sites and seasons.** Heavy metals concentrations in different seasons and in different sampling areas. Concentration in soil, root and leaves are also reported. As indicative example, Zn concentration in *Plantago major L.* is reported.

Heavy metals found in roots and leaves of the three herbaceous plants (*Plantago major L.*, *Taraxacum officinale L.* and *Urtica dioica L.*) and the leguminous *Trifolium pratense L.*, allowed us to conclude that the content of heavy metals in roots is higher than in leaves and that accumulation process of herbaceous plants does not significantly differ from that of leguminous plants: the higher the metal concentration in the soil, the higher the concentration in roots and consequently in leaves (Fig. 2).

**Figure 3:**
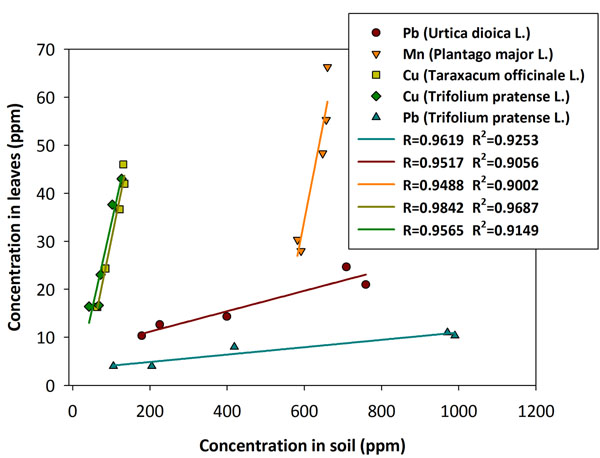
**Correlation of heavy metal concentrations in soil and leaves.** The correlation coefficient (expressed as R and R^2^) of the linear curve obtained after fitting the heavy metals concentrations in leaves against that of soil, in herbaceous and leguminous species.

We further analyzed the correlation between heavy metals content in soil and in leaves of the various vegetal species. We calculated the mean value of heavy metals concentrations in soil and leaves taking into account the values obtained in the three seasons. In this calculation we also considered all the sampling areas in order to analyze different levels of pollution. We calculated the correlation coefficients (Pearson’s correlation) between these two set of data and we considered only those metals with r ≥ 0.95. We therefore found that for *Plantago major L.* Mn has a correlation coefficient of 0.950, in *Taraxacum officinale L.* Cu has a coefficient of 0.984, in *Urtica dioica L.* Pb has a correlation of 0.952 while in *Trifolium pratense L.* Cu and Pb have coefficients of 0.956 and 0.962, respectively (Table 5 and Fig. 3).

**Table 5:** **Heavy metals mean concentration for selected herbaceous plants.** Concentrations of heavy metals contained in selected common plants. Data have been reported together with correlation coefficients in Figure 3.

	Urtica dioica L.			
**Pb**	*Mean concentration* (*ppm*)		*Standard Deviation*	

	Soil	Leaves	Soil	Leaves

SA1	708.7	24.7	180	4.7
SA2	759	21	272.2	2
SA3	399	14.3	169.2	1.5
SA4	225.3	12.7	71.1	1.5
SA5	178.7	10.3	37	1.5

				

	**Taraxacum officinale L.**			

**Cu**	*Mean concentration* (*ppm*)		*Standard Deviation*	

	Soil	Leaves	Soil	Leaves

SA1	131	46	5	9.5
SA2	134	42	15.6	3
SA3	121.3	36.7	29.7	2.5
SA4	85	24.3	37.7	1.5
SA5	63.7	16.3	36.8	7.1

				

	**Plantago major L.**			

**Mn**	*Mean concentration* (*ppm*)		*Standard Deviation*	

	Soil	Leaves	Soil	Leaves

SA1	656.7	55.3	104.7	26.8
SA2	591	28	121	6.6
SA3	647.3	48.3	118.4	21.8
SA4	660	66.3	140	22.4
SA5	582	30.3	150.3	6.4

				

	**Trifolium pratense L.**			

**Cu**	*Mean concentration* (*ppm*)		*Standard Deviation*	

	Soil	Leaves	Soil	Leaves

SA1	126.6	43	33.6	34
SA2	102.4	37.7	33.6	19
SA3	67.7	16.7	13.7	6.6
SA4	72.3	23.1	29.3	5.9
SA5	42.3	16.4	6.6	4.6

				

	**Trifolium pratense L.**			

**Pb**	*Mean concentration* (*ppm*)		*Standard Deviation*	

	Soil	Leaves	Soil	Leaves

SA1	970.7	11	242.7	4.6
SA2	990	10.3	330.3	4.7
SA3	418.3	8	159.1	3.6
SA4	204.7	4	127.8	0.1
SA5	105.3	4	23.4	2.8

## Discussion

### Heavy metals in soil

The amount of heavy metals in soil is extremely variable and these differences are more clearly emphasized if we consider different sampling areas. Different anthropogenic activities may locally alter the amount of some heavy metals, especially of those sites located near high-traffic roads. We found that the amount of some of these metals can be very high (higher than 1000 ppm for some metals) while in the control site (a non polluted park) the concentrations are relatively low. In this study we did not evaluate the effect of the various vegetal species in determining a different ‘local environment’ that we selected for analytical determination. We did not considered also the various effects of pH, temperature and other physicochemical parameters that can influence the relative heavy metals concentration. However, we found that heavy metals concentration directly correlate with the degree of pollution and, as a consequence, of anthropogenic activity in agreement with previous authors that reported that the principal source of heavy metals pollution (96% for Pb, 66% for Zn and 56% for Cu) originates from human activities [22].

### Seasonal variation of heavy metals in soil

We found a seasonal variation of heavy metals concentration in soil, that we ascribed to a different level of metal dissolution due to rainfall. In fact, during summer the rainfalls are reduced if compared to spring or autumn and high temperatures (or an increase in evapotranspiration) favour an increase of metals concentrations.

Manganese has been found almost equally distributed in all the sampling areas and this indicates that the presence of this metal in soil was not only due to anthropogenic sources (as in most polluted areas) but also to some other sources, most likely of natural origin. In fact it has been reported that Mn present in soil comes for 89% from natural sources and only for the 11% from human activities [22]. Moreover, Mn gives rise to quite complex acid-base and redox equilibrium reactions in soil, depending on conditions (temperature, soil pH and structure, humidity, etc.) leading to a bio-distribution and bio-availability difficult to analyze in details without a widespread investigation that is beyond the scope of this work.

Taking into account the seasonal distribution of heavy metals in soil and the rainfall in Rome (Fig.1) we can hypothesize that higher temperatures and reduced rainfalls may determine a higher water evaporation leading to a higher accumulation (as dry weight) of metals with respect to spring or autumn. Cu, and Pb seem to follow such behaviour, with a maximum concentration during summer (214 ppm and 1266 ppm, respectively). Zn reaches a maximum during autumn (742 ppm) and Mn follows an opposite trend showing the lowest value during summer (449 ppm). Cr and Pd do not seem to be influenced by atmospheric conditions and their concentration remain relatively low and constant all over the year (between 15 and 45 ppm for Cr and between 37 and 77 ppm for Pd). Owing to the low Pd concentration and the almost equal distribution in all the sampling areas considered, we may conclude that the eventual release of this metal from catalytic converters is therefore negligible, at least in our study. Interestingly, We also noticed the same correlation between heavy metals accumulation in soil and the concentration of some selected metals found by Cardarelli et al. in lichens collected in Rome in the same periods [23]. The same increasing trend from spring to summer may be found for Cu, Zn and Pb, with maximum concentrations during summer (47 ppm for Cu, 260 ppm for Zn and 180 for Pb); on the contrary, Mn concentration decreases showing a minimum value (32 ppm) in summer. The decrease of Mn concentration in lichens was attributed to a loose in vitality of these species, owing to the mediator effect of this metal in photosynthetic processes. Lichens are currently used as reliable bio-accumulators and bio-monitoring species to evaluate urban pollution (i.e., air quality). Since a similar behaviour was observed between air and soil pollutants, we can hypothesize the presence of a mechanism of transport from air to soil (most likely due to precipitations). However, our study suggests the presence of other mechanisms or events that should contribute to explain the reduced Mn content during summer. These events are not easily inferable and the collection of other data are needed to explain this behaviour.

### Heavy metals accumulation in plants

In our study we have considered four different vegetal species (three herbaceous and one leguminous plants) in order to investigate the feasibility of employing them as useful and simple tools to monitor environmental pollution, and in particular soil pollution by heavy metals. We therefore investigated if these plants can be selective toward specific heavy metal and in order to minimize variability in the analytical determination, we assessed the heavy metals concentration in three different seasons over the course of one solar year. From our extensive study, we found some direct correlations between the amount of heavy metals in soil and in the leaves of the selected plants (Fig. 3). Only Cu, Mn and Pb display a good linear dependence on metal concentration in soil. In particular, both *Taraxacum officinale L.* and *Trifolium pratense L.* can accumulate Cu in their leaves in a linearly dependent manner respect to soil content. Additionally, the fraction of Cu accumulated by these two species is quite high (25-40%) if compared to the amount present in soil. On the other hand, *Plantago major L.* can accumulate only small fractions of Mn (5-10%) in their leaves. *Urtica dioica L.* and *Trifolium pratense L.* are both able to accumulate Pb in their leaves even if at different percentages (10-20% for *Trifolium pratense L.* and 30-60% for *Urtica dioica L.*). For the latter two species, *Urtica dioica L.* represents the vegetal species that can accumulate the highest fraction of a dangerous heavy metal such as Pb. The higher amount of Pb in the most polluted sampling areas (near trafficked roads) is a direct consequence of anthropogenic contribution, since in 1999 Pb was still added into fuels as an additive agent.

## Conclusions

Our results demonstrate that common herbaceous and leguminous plants can be used as alternative and simple analytical tools that can be employed to monitor environmental pollution and in particular soil pollution by heavy metals. Other physicochemical parameters such as soil pH, temperature, humidity, soil texture analysis, microbiological composition and soil redox potential, to cite only a few, have to be considered in order to deeply study the metal accumulation mechanisms by plants and employ them as efficient indicators of environmental pollution. Moreover, increasing the number of vegetal species it will be possible to find better indicators for different heavy metals, and suggest a panel of common plants to employ routinely in analytical determinations for environmental pollution monitoring.

## Competing interests

The corresponding author confirms that any or all personal, employment or commercial affiliations, stock or equity interests or patent-licensing arrangements that could be considered to pose a financial conflict of interest regarding the submitted manuscript have been disclosed to the editor or in the manuscript.

## Authors' contributions

DM collected soil and plants samples for ICP-MS analysis, prepared them and helped AG to perform the analytical determinations, AG acquired and analyzed data, GO contributed to the writing of the manuscript and revision, AM organized the experimental setting, supervised the work and wrote the manuscript.
